# Dominant depressive, anxious and cyclothymic affective temperaments lower the chance of infertility treatment success

**DOI:** 10.1192/j.eurpsy.2024.1690

**Published:** 2024-08-27

**Authors:** G. Szabó, J. Szigeti F, M. Sipos, S. Varbiro, X. Gonda

**Affiliations:** ^1^Doctoral School of Mental Health Sciences, Semmelweis University; ^2^Buda Family Centre Mh Centre Department Of Psychiatry, North Centre Buda New Saint John Hospital and Oupatient Clinic; ^3^ Institute of Behavioral Sciences; ^4^Department of Obstetrics and Gynecology; ^5^Department of Psychiatry and Psychotherapy, Semmelweis University, Budapest, Hungary

## Abstract

**Introduction:**

Affective temperaments can play a significant role in the development, progression and outcome of various somatic diseases, as well as in the effectiveness of their treatment. Although infertility is influenced by both physical and psychological factors, the relationship between affective temperaments and infertility treatment success remains unexplored.

**Objectives:**

The aim of this retrospective cohort study was to assess how dominant affective temperaments influence the outcome of infertility treatments.

**Methods:**

Data was collected from a cohort of infertile women who underwent infertility treatment at an Assisted Reproduction Center in Budapest, Hungary. The study recorded treatment success defined as clinical pregnancy, detailed medical history, demographic parameters, and administered the Temperament Evaluation of Memphis, Pisa, Paris, and San Diego Autoquestionnaire (TEMPS-A). TEMPS-A scores then were classified into nondominant and dominant temperaments for each scale, based on their score being above or below the mean+2 standard deviation for the given temperament. The predictive value of dominant temperaments on assisted reproduction outcomes were analyzed by multivariate logistic regression models, using age, BMI and previous miscarriage as covariates.

**Results:**

In the cohort of 578 women who underwent infertility treatment, besides age, BMI, and previous miscarriage, dominant depressive, anxious and cyclothymic temperament decreased the odds of achieving clinical pregnancy by 85% (p=0.01), 64% (p=0.03), and 60% (p=0.050), respectively).

**Conclusions:**

The findings of this study suggest that dominant affective temperaments have a significant impact on the outcomes of infertility treatments. As a clinical consequence, creening for affective temperaments, Identifying dominant affective temperaments, stratifying high-risk patient groups, and offering personalized treatment options may enhance the likelihood of successful pregnancy and live birth for women undergoing in vitro fertilization treatment.

**Disclosure of Interest:**

None Declared

